# Emergency and critical care providers’ perception about the use of bedside ultrasound for confirmation of above-diaphragm central venous catheter placement

**DOI:** 10.1016/j.heliyon.2019.e03113

**Published:** 2020-01-07

**Authors:** Quincy K. Tran, Mark Foster, Justin Bowler, Mia Lancaster, Jennifer Tchai, Katie Andersen, Ann Matta, Daniel J. Haase

**Affiliations:** a University of Maryland School of Medicine, Department of Emergency Medicine, Baltimore, MD, USA; b University of Maryland School of Medicine, The R Adams Cowley Shock Trauma Center, Baltimore, MD, USA; c University of Maryland at College Park, College Park, MD, USA; d University of Maryland Medical Center, Baltimore, MD, USA

**Keywords:** Health profession, Medicine, Emergency medicine, Medical imaging, Central venous catheters, Above diaphragm, POCUS, Barriers

## Abstract

**Introduction:**

Chest radiography (CXR) is commonly used to confirm the proper placement of above-diaphragm central venous catheters (CVCs) and to detect associated complications. Recent studies have shown that point-of-care ultrasound (POCUS) has better sensitivity and is faster than CXR for these purposes. We were interested in documenting how often emergency medicine and critical care practitioners perform POCUS to confirm proper CVC positioning as well as their confidence in performing it.

**Methods:**

We surveyed members of our state's chapters of the College of Emergency Physicians and the Society of Critical Care Medicine between April and December 2018. Our primary outcome was the percentage of providers who would agree to perform only POCUS, forgoing CXR, for confirmation of CVC position. We performed multivariable logistic regressions to measure associations between demographic, clinical information, and outcomes.

**Results:**

One hundred thirty-six providers participated (a 25% participation rate). Their specialties were as follows: emergency medicine, 75%; critical care, 13%; and emergency medicine/critical care, 11%. Thirty-one percent would use POCUS only for CVC confirmation, while 42% were confident in performing POCUS for this purpose. Multivariable logistic regressions showed that performing more non-procedural ultrasound examinations was associated with a higher likelihood of agreeing to perform POCUS only (OR, 2.9; 95% CI: 1.3–6.3). Forty-six percent of relevant comments suggested more training to increase the use of POCUS.

**Conclusion:**

Participants in this study did not frequently use POCUS for CVC confirmation. Designers of training curricula should consider including more instruction in the use of POCUS to confirm proper CVC placement and to detect complications.

## 1 Introduction

Insertion of central venous catheters (CVCs), which allow delivery of medication and nutrients not otherwise given safely via peripheral venous catheters, is a common procedure for critically ill patients in emergency departments (EDs) and intensive care units (ICUs) [1]. Unfortunately, these catheters carry a risk of mechanical complications and infections, hampering 5%–19% of cases [2, 3, 4].

Above-diaphragm CVCs, i.e., those placed in the subclavian or internal jugular vein, present risks of misplacement (up to 14%) and iatrogenic pneumothorax (3%), which could worsen hemodynamic instabilities and delay resuscitation [2, 3]. Traditionally, chest radiography (CXR) has been considered the gold standard for confirming CVC position and detecting complications [4]. In clinical practice, after placement of an above-diaphragm CVC, the clinician must wait for CXR to confirm the tube's position and the absence of complications before the catheter can be used. Previously, different techniques were used to confirm CVC's tip position, including right atrial electrocardiography [5, 6], electrocardiogram guidance [7] and point of care ultrasound (POCUS).

In the past few years, studies have shown that point-of-care ultrasound has better efficacy than CXR in detecting above-diaphragm CVCs’ misplacement and complications [8, 9, 10, 11]. Ablordeppey and colleagues reported that, compared with CXR, POCUS reduced the time to CVC confirmation by 58 min [4], implying that treatment could commence almost 1 h sooner.

The studies mentioned above did not examine providers’ perceptions about using bedside ultrasound instead of CXR to confirm CVC placement and rule out complications. We found no reports describing the reasons that health care providers would or would not perform POCUS after placing above-diaphragm CVCs. So we designed a pilot study to assess the perceptions of medical care providers regarding the use of POCUS, their willingness to use it (forgoing CXR) to confirm CVC placement, and their perceived barriers to its use.

## 2 Methods

### 2.1 Study setting and participant selection

We sent a web-based survey to the members of our state's chapters of the College of Emergency Physicians (approximately 250 member) and the Society of Critical Care Medicine (approximately 280 members). The study period was April 1 to December 1, 2018. We sent reminders about the survey to those members four times during the study period. We kept the survey open for the specified period in order to increase participation in the study. The study was exempted by our institutional review board.

### 2.2 Development of the survey tool

The questionnaire was developed by three of the authors, who are practicing clinicians in both emergency medicine and critical care medicine. One of them is a registered diagnostic medical sonographer. The questionnaire collected demographic information, asked about respondents' clinical practice, and solicited their opinions about the use of POCUS for CVC confirmation (Appendix 1). Demographic questions had multiple-choice answers. Questions about clinical practice related to CVC insertion and opinions about the usefulness of POCUS for CVC placement were answered using Likert's scale (scores 1–5). The questionnaire was tested by 10 critical care fellows and 2 advanced practice providers (APPs). Its content was not changed after the validation phase.

### 2.3 Outcome

Our primary outcome was the percentage of participants who would perform only POCUS, forgoing CXR, to confirm proper catheter placement and the absence of complications. Our secondary outcome was the percentage of participants who were confident with using POCUS for CVC confirmation.

### 2.4 Data collection and analysis

We used descriptive data to categorize participants’ demographic characteristics and clinical practice. We dichotomized the answers using the Likert scale: the answers of “strongly agree” and “agree” (Likert scores 5 and 4) were coded as 1, and the answers of “somewhat agree,” “disagree,” and “strongly disagree” (Likert scores 3, 2, and 1) were coded as 0.

We used logistic regression to measure the association between demographics and clinical practice with our outcomes of interest. For our intention-to-treat analysis, the logistic regressions included only those participants who completely answered all questions. We initially used univariate logistic regression to examine associations between single independent variables and our outcomes of interest. Subsequently, all independent variables with a p value ≤ 0.10 were included in the multivariable logistic regressions. The goodness-of-fit of our multivariable logistic regression models was measured by the Hosmer-Lemeshow test, for which a p value > 0.05 was considered a good fit.

We analyzed the data using Sigma Plot version 13 (Systat Software, California, USA). Variables with a p value < 0.05 from the multivariable logistic regression were considered significant.

## 3 Results

### 3.1 Patient characteristics

One hundred thirty-six practitioners participated in our survey (a 26% participation rate); 127 (24% participation rate) of them answered all questions regarding clinical practice and clinical opinions and thus were included in the regression analyses. Emergency medicine was the specialty of 103 (76%) participants, while emergency medicine & critical care, and critical care were reported as the specialty of 15 (11%) and 18 (13%) participants, respectively (Table 1). The majority of participants were physicians (98%) and two-thirds (67%) were attending physicians (Table 1). Ninety participants (66%) worked in teaching facilities and the remaining 46 (34%) in non-teaching facilities.

**Table 1:** Characteristics of participants.

	N (%)
**Type of facility**∗	
Teaching	90 (66)
Non-teaching	46 (34)
**Does your institution require CXR for complications or misplacement?**∗	
Yes	109 (80)
No	8 (6)
Not sure	19 (14)
**Specialty**∗	
Emergency medicine	103 (76)
Emergency medicine and critical care	15 (11)
Critical care	18 (13)
**Health care role**∗	
Physician	133 (98)
Nurse practitioner	2 (1)
Physician assistant	1 (1)
**Level of training**∗	
Attending	91 (67)
Fellow	11 (8)
Resident	31 (23)
Other	3 (2)
**Years as attending**∗	
1‒5	34 (37)
6‒10	22 (24)
11‒20	27 (30)
>20	8 (9)
**Annual number of above-diaphragm CVCs placed**	
0‒10	43 (34)
11‒20	48 (38)
>21 **[Should “>” be changed to “≥”?]**	36 (28)
**Percentage of ultrasound-guided subclavian CVCs**	
0‒25	72 (57)
26‒50	11 (9)
51‒75	13 (10)
76‒100	31 (24)
**Percentage of ultrasound-guided IJ CVCs**	
0‒25	1 (1)
26‒50	3 (2)
51‒75	1 (1)
76‒100	122 (96)
**Annual number of non-procedure-based ultrasound examinations**	
0	5 (4)
1‒25	23 (18)
26‒50	21 (17)
>50	78 (61)
**Frequency of using ultrasound to evaluate complications**	
Always	19 (15)
Sometimes	74 (58)
Never	34 (27)
**Frequency of using ultrasound to evaluate misplacement**	
Always	26 (20)
Sometimes	62 (49)
Never	39 (31)
**Whether ultrasound is useful to rule out complications**	
Agree	106 (84)
Neutral	13 (10)
Disagree	8 (6)
**Whether ultrasound is useful to rule out misplacement**	
Agree	95 (75)
Neutral	17 (13)
Disagree	15 (12)

127 participants answered all questions regarding clinical practice and clinical opinions.

CXR, chest radiography; CVC, central venous catheter; IJ, internal jugular.

∗ 136 participants answered these demographic questions.

### 3.2 Clinical practice and opinions about POCUS and CVC confirmation

Almost all of the study participants (96%) used ultrasound guidance for insertion of CVCs. Up to 96% of participants in our study reported using POCUS for insertion of internal jugular CVC and 24% for subclavian vein CVC. Besides, using POCUS for CVC insertion, participants in our study also used POCUS for non-procedural bed side examination. Up to 61% of participants performed >50 non-procedure-based ultrasound examinations every year, e.g., Focal Assessment with Sonography in Trauma (FAST) and assessment of cardiac function.

Nineteen (15%) participants reported that they always use ultrasound to evaluate above-diaphragm CVC complications, compared with 34 (27%) who reported that they never use ultrasound for this purpose (Table 1). Similarly, 26 (20%) always use ultrasound to look for misplacement whereas 39 (31%) never use ultrasound for this purpose.

When asked about the usefulness of ultrasound to check for above-diaphragm CVC complications and misplacement, 106 (84%) and 95 (75%) participants, respectively, agreed that it is useful.

### 3.3 Outcomes

Thirty-one (24%) participants reported that they use only POCUS to confirm the absence of CVC complications and misplacement (Table 2). Interestingly, 42 (33%) participants reported that they were confident in using ultrasound to detect complications and misplacement.

**Table 2:** Agreement with using ultrasound only and confidence in its use for complications and misplacement of above-diaphragm CVCs.

	N (%)
**Use ultrasound only, no CXR, for complications**	
Agree	47 (37)
Neutral	24 (19)
Disagree	56 (44)
**Use ultrasound only, no CXR, for misplacement**	
Agree	40 (31)
Neutral	26 (20)
Disagree	61 (48)
**Use ultrasound only, no CXR, for both complications and misplacement**∗	31 (24)
**Confidence of using ultrasound to detect CVC complications**	
Confident	64 (50)
Somewhat	23 (18)
Slightly	19 (15)
Not confident	21 (17)
**Confidence of using ultrasound to detect CVC misplacement**	
Confident	52 (41)
Somewhat	29 (23)
Slightly	16 (12)
Not confident	30 (24)
**Confidence of using ultrasound to detect both complications and misplacement**∗∗	42 (33)

CXR, chest radiography; CVC, central venous catheter.

∗ The Likert scale answers of “strongly agree” and “agree” were categorized as “agree”; all others were categorized as “not agree.”

∗∗ The likert scale answers of “strongly confident” and “confident” were categorized as “confident”; all others were categorized as “not confident.”

Multivariable logistic regression, after adjusting for relevant factors (Appendix 2) showed that one clinical practice—performing a high number of non-procedure ultrasound examinations each year—was significantly associated with a higher likelihood of agreeing to use only POCUS to detect above-diaphragm CVC complications and misplacement (OR, 2.8; 95% CI: 1.3–6.3 [p = 0.003]) (Table 3).

**Table 3:** Multivariable Logistic Regressions to Assess Associations Between Participants’ Characteristics, Clinical Practices, and Study Outcomes. Only significant factors were reported.

	Univariate Logistic Regression			Multivariable Logistic Regression		
	OR	95% CI	p	OR	95% CI	p
**Primary Outcome: using ultrasound ONLY to rule out both complications and misplacement**^a^						
Annual number of US examinations	2.9	1.4–6.2	<0.001	2.8	1.3–6.3	0.003
**Secondary Outcome: confidence of using ultrasound to rule out both complications and misplacement**^b^						
Specialty: Emergency Medicine	0.4	0.2–0.9	0.035	0.01	0.0001–0.22	0.001
Use US for misplacement: Never	0.03	0.04–0.2	0.001	0.02	0.001–0.3	0.001

Only factors that showed clinically significant association are reported.

CI, confidence interval; OR, odds ratio; US, ultrasound.

a Hosmer-Lemeshow test results: χ [2] = 1.6, p = 0.98.

b Hosmer-Lemeshow test results: χ [2] = 3.68, p = 0.89.

Being in the specialty of emergency medicine was associated with significantly less confidence in the use of ultrasound to detect complications and misplacement (OR, 0.01; 95% CI: 0.0001–0.2 [p = 0.001]). Participants who said they never use ultrasound to assess for misplacement had a lower likelihood of using ultrasound to detect both misplacement and complications (OR, 0.02; 95% CI: 0.001–0.3 [p = 0.001]) (Table 3).

### 3.4 Suggestions for increasing the use of ultrasound

We collected 78 free-text comments from our participants; 74 of them were relevant regarding interventions that could increase the frequency of POCUS use for CVC confirmation (Table 4). Most of the comments (45%) suggested more training of providers. Twenty (25%) expressed interest in more evidence supporting the use of ultrasound (Table 4).

**Table 4:** Suggestions about providers’ perceived barriers and how to increase the use of POCUS after placement of above-diaphragm central venous catheters∗.

Suggestions	N (%)†
More training	36 (46)
More evidence	20 (26)
Institutional or professional organizational guidelines	17 (22)
More practice	13 (17)
Technically difficult	11 (14)
Other	4 (5)

POCUS, point of care ultrasound.

∗ The survey collected 78 comments; 74 of them (95%) were relevant responses. Some comments contained more than one suggestion.

† Percentages are based on the number of relevant responses.

## 4 Discussion

The participants in our pilot study do not use ultrasound frequently to detect complications and misplacement after insertion of above-diaphragm CVCs. Only 40% of them reported confidence in using ultrasound for those purposes. Most participants suggested that more training would increase their likelihood of using only POCUS to confirm proper CVC position and the absence of complications.

Different techniques had been used to confirm the position of CVCs' tips. When compared with CXR, intra-atrial electrocardiogram resulted in no catheter tip locations within the heart (0/25) vs. 56% [14/25, (p < .0001)] [5]. However, a subsequent study using intra-atrial electrocardiogram guidance only showed 92% of CVCs' tips were correctly positioned [6]. On the other hand, electrocardiogram guidance resulted in No malposition of CVCs’ tips [7].

Based on a meta-analysis of 15 studies, Ablordeppey et al [4] calculated that POCUS has a pooled sensitivity of 0.82 (95% CI: 0.77–0.86) and specificity of 0.98 (95% CI: 0.97–0.99) for detection of catheter malposition. Although there has not been any comparison study between POCUS and other modalities for CVCs’ tip confirmation, POCUS can also be used to detect pneumothoraces while electrocardiogram guidance still needs CXR. Therefore, POCUS can be more cost effective [12], as we discussed further below.

Among CVC-related complications, Nayeemuddin and colleagues reported that pneumothoraces occurred in approximately 3% of radiologically guided tunneled CVCs [3] and could be life-threatening. Ablordeppey et al reported that the pooled sensitivity and specificity for POCUS were both almost 100% for detection of pneumothoraces and that CXR detected only 83% of the pneumothoraces in the articles they reviewed [4]. In addition, POCUS required an average of 5.6 min to confirm the position of the CVC, compared with 64 min for CXR [4].

Several recent studies documented the benefits and cost-effectiveness [12] of using POCUS to detect complications and misplacement of above-diaphragm CVCs [4, 8, 9, 10, 11, 12] in relation to CXR. Woodlawn and associates determined the billable cost of one chest film for central line confirmation to be approximately USD 475,^12^ so the use of an alternative confirmation process would be cost saving for medical facilities and insurers, assuming that cost for POCUS would be low as it would be part of the central line bundle.

Despite the benefits of POCUS, the percentage of participants in our study who would use it as the only confirmation modality after CVC insertion was low. Further study with a larger group of respondents is needed to calculate the incidence and elucidate reasons for the slow adoption of POCUS in this clinical scenario. Barriers to the use of POCUS use after CVC placement are suggested by the responses listed in Table 4. The need for more training was mentioned often. Training curricula have traditionally emphasized ultrasound-guided CVC placement, but not detection of complications and misplacement. For example, the 2017 Canadian Internal Medicine Ultrasound Group recommended the use of POCUS for CVC placement in the core curriculum for internal medicine residents, but it did not clearly mention its follow-up confirmatory use [13]. It should be easy to expand existing training programs to include the use of POCUS after catheter insertion because additional resources would not be required. Therefore, we suggest that program directors incorporate the use of POCUS to detect complications and misplacements after CVC insertion into standard curricula.

The second most common request from our participants was for “more evidence” supporting the efficacy of the use of POCUS to detect complications and misplacement after CVC insertion. Previous reports of the use of ultrasound for CVC confirmation were not from randomized studies [4]. It remains unclear whether a randomized study between POCUS and CXR for CVC confirmation is ethical because of the compelling evidence about the efficacy of POCUS for this purpose. While we await further studies, information about the risks and benefits of using POCUS for CVC placement confirmation can be incorporated into training sessions, which would increase providers’ awareness and willingness to use it.

Almost all of our participants reported using ultrasound guidance for placement of internal jugular vein CVCs; this practice has been documented as significantly reducing the rate of complications [14]. In a recent multicenter retrospective study of almost 11,000 CVCs, 57% of which were placed in the internal jugular vein [15], mechanical complications occurred in only 1.1% of cases (among them, pneumothoraces in 0.2% and bleeding in 0.8%). At institutions with low rates of complications associated with CVC placement, it is questionable if CXR is necessary or sensitive enough. In fact, POCUS, with its good sensitivity and specificity, looks like an excellent alternative that can reduce time to treatment and its cost.

### 4.1 Limitations

Our pilot study was, to our knowledge, the first one to investigate EM and CC providers' use of POCUS for confirmation of proper CVC placement. It provides initial information for further studies into the use of POCUS for this purpose. For example, a study built on pre- and post-training assessments would document changes in health care providers’ use of POCUS after CVC placement and their confidence in it. Our study also highlighted the need for clinical guidelines from professional societies regarding the use of POCUS for central line confirmation.

Admittedly, our study has major limitations. First, the survey had a low participation rate, so the information it gathered might not be representative of the other health care providers in the collaborating organizations or in the hospitals in our state. Also, the number of APPs who completed our survey was low; future studies should do a better job of bringing this professional group into the realm of observation because they insert CVCs in many clinical settings [16]. We also included all surveys with complete data for analysis, assuming each survey came from a unique participant. Finally, we did not ask about the numbers of complications and misplacements that each participant has experienced. Those difficult clinical scenarios might have affected medical care providers’ decisions about using POCUS.

## 5 Conclusion

The emergency medicine and critical care specialists who participated in our survey do not frequently use and are not confident in using POCUS to detect complications or misplacement after insertion of a central venous catheter. Many of them suggested more training in the technique so as to increase their willingness to use ultrasound as a confirmatory tool. We recommend the expansion of training curricula beyond ultrasound-guided CVC placement to its use to confirm proper positioning and the absence of complications following above-diaphragm central venous catheterization.

## Declarations

### Author contribution statement

Quincy Tran: Conceived and designed the experiments; Performed the experiments; Analyzed and interpreted the data; Contributed reagents, materials, analysis tools or data; Wrote the paper.

Justin Bowler, Jennifer Tchai, Mia Lancaster: Performed the experiments; Contributed reagents, materials, analysis tools or data; Wrote the paper.

Mark Foster, Katie Andersen, Ann Matta, Daniel Haase: Conceived and designed the experiments; Analyzed and interpreted the data; Wrote the paper.

### Funding statement

This research did not receive any specific grant from funding agencies in the public, commercial, or not-for-profit sectors.

### Competing interest statement

The authors declare no conflict of interest.

### Additional information

Supplementary content related to this article has been published online at https://doi.org/10.1016/j.heliyon.2019.e03113.

## Appendix A Supplementary data

2019_10_11 Supplementary file 01_V1 2019_10_11 Supplementary file 01_V1
