# Supplementary material for: Emergency and critical care providers’ perception about the use of bedside ultrasound for confirmation of above-diaphragm central venous catheter placement
Source: Heliyon. 2020 Jan 7;6(1):e03113. doi: 10.1016/j.heliyon.2019.e03113 (PMC7002808; doi:10.1016/j.heliyon.2019.e03113)
Supplement: 2019_10_11 Supplementary file 01_V1 [file mmc1.docx]

**Appendix 1**. Survey Tool to Assess Emergency Medicine and Intensivists About Their Opinions Regarding The Use of Point of Care Ultrasound for Above Diaphragm Central Venous Line

**Survey Title: Use of Ultrasound for Above Diaphragm Central Venous Line Placement**

| **We are conducting a survey to evaluate your current practice in using bedside ultrasound to evaluate placement and complications after insertion of a central venous line (CVL) placed above the diaphragm. The central venous lines referred to in this survey are all above diaphragm such as subclavian or internal jugular CVLs.**  **Examples of CVL complications include: pneumothorax, hemothorax Example of CVL misplacement include: arterial, wrong venous vessel**   1. After central venous line (CVL) placement, does your institution require a chest x-ray prior to CVL use to evaluate for complications?   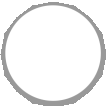 Yes 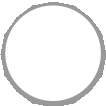 No  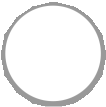 Do not know   1. After CVL placement, does your institution require a chest x-ray prior to CVL use to evaluate for misplacement?   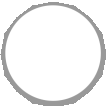 Yes 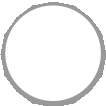 No  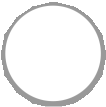 Do not know   1. At what type of health care facility do you currently work?   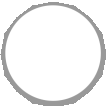 Teaching  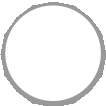 Non-teaching (non-academic, no residents)   1. What is your role in patient care?   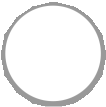 Attending 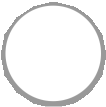 Nurse Practitioner  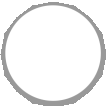 Fellow 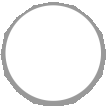 Physician Assistant  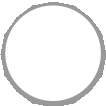 Resident |
| --- |

1. How many years have you been practicing medicine since graduating medical school?


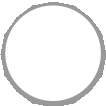
 0


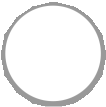
 1 - 10


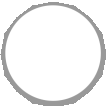
 11 - 20


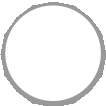
 21+

1. What is your specialty?


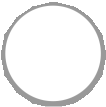
 Emergency Medicine
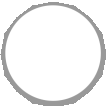
 Critical Care


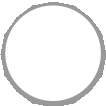
 Emergency Medicine-Critical Care
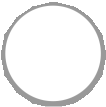
 Other

1. How many CVL placements do you perform each year?


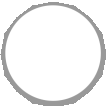
 0


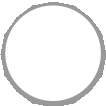
 1 - 10


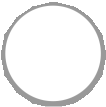
 11 - 20


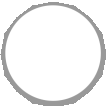
 21+

1. How many ultrasound scans do you perform each year?


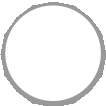
 0


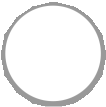
 1 - 10


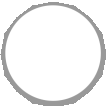
 11 - 20


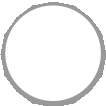
21+

1. To what extent do you agree with the following statements?

Ultrasound can be used to rule out CVL complications.

Ultrasound can be used to rule out CVL misplacement.

If it were an option, I would use ultrasound only (no chest x-ray) to evaluate for CVL complications.

If it were an option, I would use ultrasound only (no chest x-ray) to evaluate for

CVL misplacement.

Strongly Disagree Disagree

Neither Disagree

nor Agree Agree Strongly Agree

1. How confident are you in your ability to…

Rule out CVL complications by using ultrasound?

Rule out CVL misplacement by using ultrasound?

Not at all confident Slightly confident

Somewhat confident

Moderately

confident Very confident


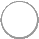

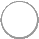

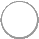

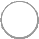

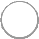

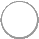

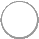

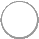

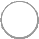

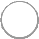

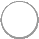

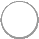

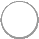

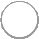

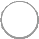

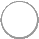

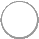

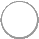

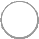

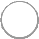

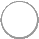

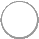

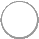

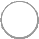

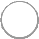

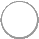

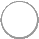

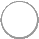

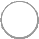

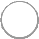

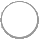

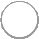

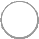

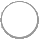

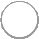

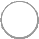


1. In your current practice, do you use ultrasound to evaluate for CVLcomplications?

Always Sometimes Never

1. In your current practice, do you use ultrasound to evaluate for CVLmisplacement?

Always Sometimes Never

1. What would make you feel more comfortable with using an ultrasound only to evaluate for CVL complications/ CVL misplacement? (Free text)

**Appendix 2**. Results of Univariate and Multivariable Logistic Regressions Assessing Association Between Emergency Medicine or Critical Care Providers’ Demographic and Clinical Factors With Outcomes.

|  | **Univariate Logistic Regression** | | | **Multivariable Logistic Regression** | | | |
| --- | --- | --- | --- | --- | --- | --- | --- |
|  | OR | 95% CI | p | OR | 95% CI | p | |
| **Using ultrasound ONLY to rule out misplacement and complications**^a^ | | | | | | | |
| Institutional policy requiring US | 0.9 | 0.3‒2.4 | 0.84 | NA | NA | | NA |
| Type of facility | 0.78 | 0.3‒1.9 | 0.58 | NA | NA | | NA |
| Specialty: EM | 2.25 | 0.7‒7.1 | 0.14 | NA | NA | | NA |
| Specialty: EMCC | 0.44 | 0.09‒2.1 | 0.26 | NA | NA | | NA |
| Specialty: CC | 0.53 | 0.11‒2.5 | 0.40 | NA | NA | | NA |
| Level of training: Fellow or resident | 1.3 | 0.5‒2.9 | 0.58 | NA | NA | | NA |
| Level of training: Attending | 0.78 | 0.3‒1.8 | 0.58 | NA | NA | | NA |
| Level of training: APP* | NA | NA | NA | NA | NA | | NA |
| Years as attending | 0.79 | 0.56‒1.1 | 0.18 | NA | NA | | NA |
| Number of above-diaphragm CVCs inserted annually | 0.99 | 0.6‒1.7 | 0.99 | NA | NA | | NA |
| Percentage of SCV under US | 1.4 | 0.99‒1.8 | 0.052 | 1.3 | 0.9‒1.8 | | 0.17 |
| Percentage of IJ under US | 1.1 | 0.4‒3.3 | 0.8 |  |  | |  |
| Number of US examinations | 2.9 | 1.4‒6.2 | <0.001 | 2.8 | 1.3‒6.3 | | 0.003 |
| Ultrasound is useful to detect complications: Agree | 3.6 | 0.78‒16.3 | 0.059 | 1.3 | 0.2‒8.3 | | 0.75 |
| Ultrasound is useful to detect misplacement: Agree | 2.8 | 0.89‒8.7 | 0.056 | 2.1 | 0.5‒9.1 | | 0.33 |
| Use US for complications: Always | 1.5 | 0.5‒4.4 | 0.44 | NA | NA | | NA |
| Use US for complications: Sometimes | 1.2 | 0.5‒2.7 | 0.69 | NA | NA | | NA |
| Use US for complications: Never | 0.58 | 0.2‒1.6 | 0.27 | NA | NA | | NA |
| Use US for misplacement: Always | 2.9 | 1.2‒7.4 | 0.02 | 2.1 | 0.8‒6.0 | | 0.14 |
| Use US for misplacement: Sometimes | 0.8 | 0.3‒1.9 | 0.64 | NA | NA | | NA |
| Use US for misplacement: Never | 0.45 | 0.2‒1.223 | 0.10 | 1.3 | 0.3‒4.5 | | 0.67 |
| **Confidence of using ultrasound for misplacement and complications**^b^ | | | | | | | |
| Institutional policy requiring US | 1.2 | 0.5‒3.1 | 0.66 | NA | NA | | NA |
| Type of facility | 0.6 | 0.3‒1.4 | 0.24 | NA | NA | | NA |
| Specialty: EM | 0.4 | 0.2‒0.9 | 0.035 | 0.01 | 0.0001‒0.22 | | 0.001 |
| Specialty: EMCC | 2.6 | 0.9‒7.8 | 0.08 | 0.03 | 0.005‒2.0 | | 0.09 |
| Specialty: CC | 1.8 | 0.6‒5.9 | 0.30 | NA | NA | | NA |
| Level of training: Resident or fellow | 0.96 | 0.4‒2.1 | 0.43 | NA | NA | | NA |
| Level of training: Attending | 1.03 | 0.5‒2.3 | 0.93 | NA | NA | | NA |
| Level of training: APP* | NA | NA | NA | NA | NA | | NA |
| Years as attendings | 0.7 | 0.5‒0.96 | 0.02 | 0.86 | 0.5‒1.5 | | 0.57 |
| Number of above-diaphragm CVCs inserted annually | 1.03 | 0.7‒1.6 | 0.88 | NA | NA | | NA |
| Percentage of SCV under US | 1.4 | 1.05‒1.8 | 0.02 | 1.1 | 0.71‒1.6 | | 0.74 |
| Percentage of IJ under US* | NA | NA | NA | NA | NA | | NA |
| Number of US examinations | 2.1 | 1.2‒3.6 | 0.002 | 2.4 | 0.98‒5.9 | | 0.37 |
| Ultrasound is useful to detect complications: Agree | 13 | 1.6‒97 | 0.001 | 3.8 | 0.2‒91 | | 0.36 |
| Ultrasound is useful to detect misplacement: Agree | 23 | 3‒100+ | 0.001 | 21 | 0.7‒100+ | | 0.24 |
| Use US for complications: Always | 5.9 | 2.1‒17 | 0.11 | NA | NA | | NA |
| Use US for complications: Sometimes | 1.9 | 0.9‒4.3 | 0.08 | 1.7 | 0.3‒9.4 | | 0.52 |
| Use US for complications: Never* | NA | NA | NA | NA | NA | | NA |
| Use US for misplacement: Always | 4.6 | 1.9‒11 | 0.11 | NA | NA | | NA |
| Use US for misplacement: Sometimes | 1.9 | 0.9‒4.0 | 0.19 | 0.3 | 0.07‒1.5 | | 0.13 |
| Use US for misplacement: Never | 0.03 | 0.04‒0.2 | 0.001 | 0.02 | 0.001‒0.3 | | 0.001 |

^a^Hosmer-Lemeshow test results: χ^2^=1.6, p=0.98

^b^Hosmer-Lemeshow test results: χ^2^=3.68, p=0.89

*Too few variables, so logistic regression was not performed.

APP, advanced practice provider; CC, critical care; CI, confidence Interval; CVC, central venous catheter; EM, emergency medicine; EMCC, emergency medicine and critical care; IJ, internal jugular vein catheter; NA, not included in the multivariable logistic regression; OR, odds ratio; SCV, subclavian vein catheter; US, ultrasound
